# How adaptive social robots influence cognitive, emotional, and self-regulated learning

**DOI:** 10.1038/s41598-025-91236-0

**Published:** 2025-02-24

**Authors:** Helene Ackermann, Anna L. Lange, Verena V. Hafner, Rebecca Lazarides

**Affiliations:** 1 Department of Educational Sciences, Universität Potsdam, Karl-Liebknecht-Straße 24/25, 14476 Potsdam, Germany; 2 Science of Intelligence, Research Cluster of Excellence, Marchstraße 23, 10587 Berlin, Germany; 3 Department of Computer Science, Humboldt-Universität zu Berlin, Unter den Linden 6, 10099 Berlin, Germany

**Keywords:** Human behaviour, Information technology

## Abstract

As educational environments become more diverse, adaptive technologies like social robots hold promise for providing individual support to learners. This study investigated the role of adaptive teaching of a robot on students’ learning outcomes, emotions, and self-regulated learning (SRL). A total of 120 participants (aged 18–60 years, *M*_age_ = 30.25, *SD*_age_ = 10.06, 64.3% female) engaged in an interactive vocabulary learning task with varying guidance levels by the robot. Control conditions included fixed guidance, offering either simple guidance with no hints (condition 1) or enhanced guidance, constantly adding hints (condition 2). In the adaptive conditions, the robot adjusted the number of hints based on learners’ recent performance and enjoyment (condition 3) or additionally personalized hints to specific mistakes (condition 4). The results showed no direct or indirect effects of adaptive guidance on task performance or cognitive learning compared to fixed guidance. Instead, adaptive guidance significantly reduced on-task enjoyment compared to enhanced guidance, suggesting that unexpected variability in robot behavior may negatively affect emotional learning experience. However, personalized adaptive guidance increased certain SRL behaviors, indirectly benefiting learning outcomes. These findings highlight the need for further refinement of adaptive social robots to better meet individual learner needs and optimize outcomes.

**Supplementary Information:**

The online version contains supplementary material available at 10.1038/s41598-025-91236-0.

## Introduction

Classroom heterogeneity is an inherent characteristic of today’s educational environments. Classrooms are diverse, with students exhibiting a wide range of cognitive abilities, backgrounds, and emotional needs^1^. While this diversity can enhance students’ social-emotional skills and foster an inclusive learning culture^2^, it also presents challenges for school systems and teachers who are tasked with tailoring instruction to meet students’ individual needs^3^. Adaptive teaching is demanding and complex, requiring teachers to combine technical skills and creative decision-making to address these varied needs effectively^4^. In addition, teachers often face large class sizes, limited time, and a high cognitive load^5^, which also contribute to this difficulty. To support teachers in navigating this complexity, there is a growing need for research into adaptive teaching approaches that can offer practical assistance. Advances in technology, particularly adaptive learning systems, hold promise by providing personalized support that adjusts to learners’ evolving needs in real-time^6,7^. This study aims to contribute to this research field by examining how adaptive teaching can be embedded into technology to address the diverse needs of different learners.

Adaptive teaching is a critical component of successful learning and a key competency of effective teachers^8^. Corno and Snow conceptualized it as “teaching that arranges environmental conditions to fit learner differences”^9^. It requires balancing technical and creative skills, as teachers must decide in real-time whether students need more or less support or challenge^10^. To provide quality education, teachers must flexibly adjust their practices, instructional methods, and communication to meet diverse student needs^11^. The level of guidance should vary from minimal to intensive based on learning complexity, prior knowledge, and individual needs^12^. This approach aligns with scaffolding, a dynamic, temporary support that teachers provide by adjusting assistance (contingency), gradually reducing it (fading), and ultimately shifting responsibility to the learner (transfer)^13^. Scaffolding is theoretically grounded in Vygotsky’s concept of a zone of proximal development (ZPD), the gap between what a learner can achieve independently and with guidance^14^. Helping students to learn within their ZPD by individually adapting the level of guidance can help limit cognitive overload, which cognitive load theory^15^ suggests is crucial for effective learning by enabling students to focus on essential tasks.

Research has shown that the ability to teach adaptively improves the quality of instruction, which in turn has a positive effect on student achievement^8^. Adaptive teaching has further been shown to positively improve students’ conceptual understanding^12^ while also fostering positive emotions and reducing negative emotions when support is tailored to individual performance rather than provided at fixed levels^16,17^. This aligns with assumptions from control-value theory presented by Pekrun, which suggests that students’ emotional experiences during learning are influenced by cognitive appraisals, thus, by the perceived control they have over their performance and the value they assign to the task^18^. When instructional support is tailored to individual needs, students are likely to feel more in control and view the task as more valuable, resulting in increased positive and decreased negative emotions. High levels of positive, activating achievement emotions, such as enjoyment, in turn, positively affect the availability of cognitive resources like attention and contribute to the effective use of learning strategies, ultimately enhancing students’ performance^19^. Further, adaptive teaching can improve metacognitive learning activities and self-regulated learning (SRL) of students^13^. SRL is defined as learners’ ability to actively manage their own learning process through setting goals, monitoring performance, and changing strategies if needed^20^. According to Zimmerman’s model of SRL, it involves a cyclical, three-phase process of forethought (goal-setting and planning), performance (monitoring and strategy use), and self-reflection (evaluating outcomes and adjusting strategies)^20^. Adaptive scaffolding can be designed to enhance SRL^21^, which again helps students optimize their learning and improve learning outcomes^22^.

Scaffolding through adaptive guidance to increase SRL and metacognitive activity is frequently used in new learning technologies^21,23,24^. One example is intelligent tutoring systems (ITS), which provide personalized learning by adapting to learners’ cognitive and psychological states^25^. For instance, Cognitive Tutors^26^, an ITS for high school mathematics, offers interactive support by delivering step-by-step feedback, specific hints for common errors, and on-demand guidance^26^. Embedding prompts and reflective questions in intelligent learning environments is an effective strategy to foster reflection and SRL, guiding students toward their learning goals^24^. However, adaptive teaching in technology extends beyond reflective questioning. Gerard and colleagues identified various types of adaptive guidance in automated systems, categorizing them as simple or enhanced^27^. Simple guidance provides immediate correctness feedback, while enhanced guidance includes features that promote deeper learning and self-monitoring^27^. Enhanced guidance, by encouraging reflection and knowledge integration, has been shown to improve learning, particularly for students with lower prior knowledge^27^. These findings suggest that not all students may benefit equally from adaptive scaffolding focused solely on cognitive learning by providing prompts to deepen thinking. In particular, high-performing students may not experience the same learning gains as those with lower prior knowledge, indicating that cognitive prompts alone may be insufficient. Such findings highlight the need for adaptive systems that also support students on motivational-affective levels – a perspective that is increasingly acknowledged in research^28,29^. This underscores the complexity of modeling human adaptivity in technological systems, which simultaneously integrates cognitive, motivational, and emotional support to effectively personalize learning^6^.

Building on the growing interest in adaptive educational technologies, researchers see social robots as having particular potential for implementing automated adaptive teaching in education settings^30^. Their physical presence has been shown to increase students’ engagement and learning as compared to solely on-screen technologies^31–33^. With the ability to include nonverbal behavior, social robots can increase immediacy and promote a more natural interaction^31^, making them well-suited to model human-like teaching behavior. As a result, social robots are being increasingly explored in educational research, particularly in early childhood education, language learning, physical skills training, and social skills development^31^. For instance, in language learning, social robots that engage students through social dialogue and supportive behaviors rather than solely focusing on knowledge transfer have been demonstrated to positively impact students’ learning performance^34^. Further, Schodde and colleagues introduced an “adapt-and-explain” strategy for social robots in language learning, showing that adapting interactions based on the learner’s cognitive and affective state and providing verbal explanations of these adjustments enhanced engagement and improved learning gains^35^.

However, despite these promising outcomes, the research into adaptive social robots in education is still in its early stages, and findings are mixed. While some studies indicate that social robots can improve engagement and learning^34–37^, others show limited or no significant benefits. For instance, Gao and colleagues found that participants in a robot-supported learning scenario performed better and preferred a robot exhibiting varied supportive behaviors over one that personalized its behavior by converging on a specific type of support over time, suggesting that rigid personalization may not always be beneficial for learning outcomes^38^. Similarly, Donnermann and colleagues found that while an adaptive robotic tutor significantly improved students’ exam performance compared to those who did not receive tutoring, it did not yield additional benefits over a non-adaptive version in terms of learning gains and motivation^39^. These findings highlight the complexity of designing adaptive social robots and point to several key research gaps that need to be addressed. First, there is a gap in understanding how adaptive strategies can be optimized to provide meaningful and individualized support across diverse learning contexts. Second, many existing studies face methodological limitations, such as small sample sizes and short intervention periods^40^, which limit the generalizability of findings. Third, the transferability of complex human teaching methods – such as scaffolding – to artificial agents remains a challenging area^35^, as it is unclear how robots can replicate these techniques in a way that preserves their educational effectiveness. These gaps emphasize the need for continued research to determine how and when adaptive social robots can effectively support diverse learners across various educational settings.

To address these research gaps, the present study investigated the impact of a social robot’s adaptive teaching behavior, implemented as varying levels of guidance, on students’ learning outcomes. Specifically, we examined how the robot’s different levels of adaptivity influence students’ task performance, cognitive learning, emotional experiences, and SRL behaviors while controlling for covariates such as age and cognitive ability. Referring to theoretical assumptions from Pekrun’s control-value theory and Zimmerman’s SRL model, we further examined whether emotional experiences and SRL behaviors mediate the effects of adaptive guidance on students’ task performance and cognitive learning. To address our research questions, we conducted an experiment where participants completed a cognitive learning task with a social robot acting as their teacher, providing instructions and guidance in the form of (meta-)cognitive hints. In condition 1, the robot provided simple guidance, only offering feedback on the correctness of steps. In condition 2, the robot presented enhanced guidance by additionally providing hints after every incorrect action. In the adaptive condition 3, the robot adjusted the amount of guidance by decreasing or increasing the number of hints based on participants’ current performance and on-task enjoyment. Lastly, in condition 4, the robot provided personalized adaptive guidance, adapting both the number of hints and tailoring the feedback to specific mistakes and individual learning progress. We hypothesized that adaptive teaching (condition 3) would positively influence cognitive learning and task performance (H1) compared to the non-adaptive control conditions (conditions 1 and 2). We further hypothesized that the robot’s adaptivity would increase on-task enjoyment while reducing frustration and boredom (H2a) and that these emotional experiences would mediate the positive relation between adaptive teaching and learning outcomes (H2b). Additionally, we expected adaptive teaching to enhance SRL behaviors (H3a), which would also mediate the positive relation between adaptive teaching and learning outcomes (H3b). Lastly, condition 4 was included to exploratorily investigate differences in learning outcomes, emotional experiences, and SRL behaviors between basic adaptive (condition 3) and personalized adaptive guidance (condition 4).

## Methods

### Sample description

A total of 120 participants participated in the study, aged between 18 and 60 years (*M* = 30.25, *SD* = 10.06). The sample was predominantly female, with 77 participants (64.3%) identifying as female, 40 participants (33.3%) as male, two participants (0.2%) as gender diverse, and one participant (0.1%) preferred not to state a gender. The majority of the participants were university students (65.0%). A total of 101 participants (84.2%) were native German speakers, with the remaining participants speaking a variety of languages, including Russian (*n* = 5), Arabic (*n* = 2), Spanish (*n* = 2), Ukrainian (*n* = 2), and other languages (*n* = 8). A power analysis conducted using G*Power^41^, based on a medium effect size (*f²* = 0.15), an alpha level of 0.05, and a power level of 0.80, indicated that a minimum of 92 participants was required. The sample size was increased to 120 to ensure balanced group sizes and account for potential exclusions. Criteria for participation were very good knowledge of German, being of legal age (≥ 18 years), and no previous knowledge of Swahili. Participants were randomly assigned to one of the four experimental conditions, with 30 participants per condition. To control for potential biases, participants’ prior experience with humanoid robots was assessed, revealing no significant differences between conditions (*χ²(*3) = 6.43, *p* = .093), ensuring balanced groups. Prior to participation, informed consent was obtained from all participants. The study was performed in line with the principles of the Declaration of Helsinki and received approval from the University of Potsdam Ethics Committee (number 78/2023).

### Procedure

**Fig. 1:**
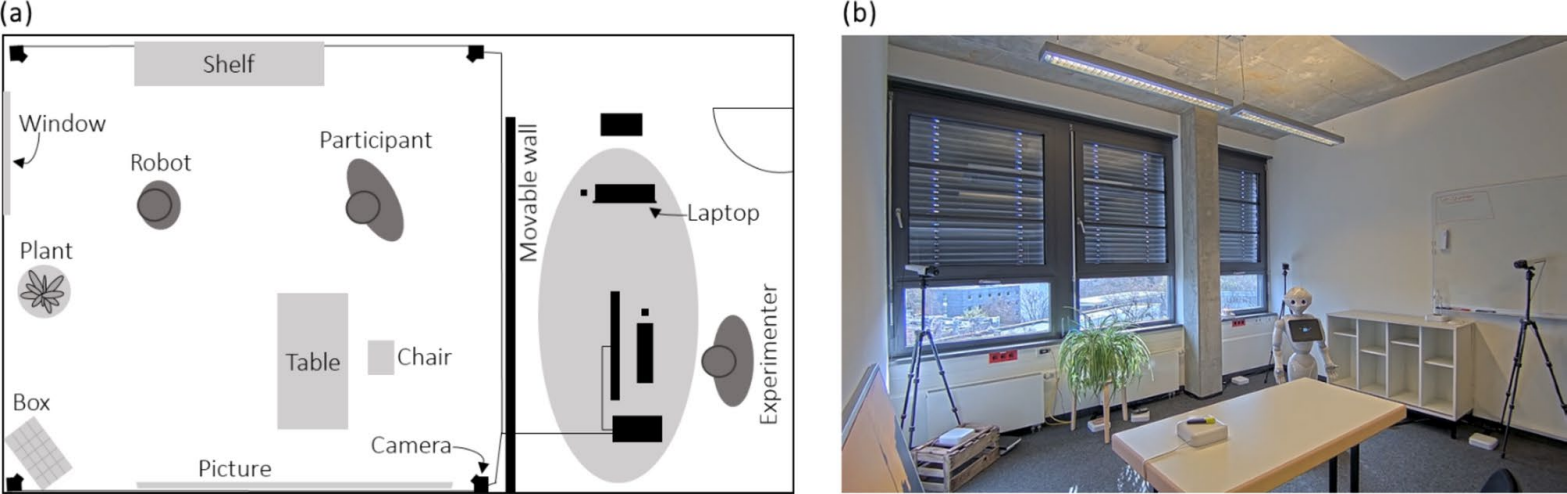
Visualization of the experimental room setup. The room was arranged for a one-on-one interaction between the participant and a Pepper robot. A movable wall separated the experimenter from the participant. Four cameras were used to observe the interaction, one in each corner of the room. The robot remained stationary but was able to rotate and track the participant as they moved around the room freely. White boxes, marking possible object placement spots, were distributed throughout the room. **(a)** Sketch of experimental set-up from above, and **(b)** photo of the interaction area taken after the session, with objects already placed.

The participants were recruited through the digital participant tool of the University of Potsdam, and the study was advertised via posters. Each participant scheduled a 90-minute individual slot and interacted one-on-one in a learning scenario with a Pepper robot. After a brief introduction, they completed a pre-test on a laptop, which assessed their sociodemographic information and general cognitive ability level. Afterwards, participants engaged in an interactive vocabulary learning game with the Pepper robot acting as their teacher. In the game, participants had to place objects in certain predefined locations in the experimental room (see Fig. 1). Participants were instructed about correct placements by the robot, who communicated this information in Swahili, a language unfamiliar to the participants. Swahili was selected due to its grammatical structure, its unfamiliarity to participants, and its phonetic compatibility with the robot’s German language package, ensuring clear pronunciation. The participants task was to decode the provided information and learn the vocabulary to understand and follow the object placement instructions. The learning session ended either when all twelve objects were placed at their correct locations or when the experimental time of 30 min elapsed. After the interaction, participants completed a post-test on the laptop, which included a vocabulary test to assess their cognitive learning outcomes.

Before starting the game, Pepper introduced itself and explained the task to the participants. It described the game mechanics, explaining that participants would need to place a total of twelve objects (e.g., bottle, pen, book) at specific locations in the room. Possible placement spots were identified by 16 small white boxes scattered throughout the room, and participants were instructed to only place objects on these designated areas to receive feedback from the robot. Each object had exactly one correct location. Participants could freely choose which object to position next but had to place it on a start position first to receive instructions on where to place it. The instructions followed a consistent three-word structure consisting of the object, preposition, and target location (e.g., “chupa – kwenye – rafu”, meaning “bottle – on – shelf”). Participants would then have to place the object (i.e., bottle) on the white box located on the shelf. After each placement, Pepper provided feedback in German, indicating whether it was correct or incorrect. In cases of an incorrect placement, such as placing the bottle under the table, Pepper would add information about the current position (i.e., “chupa – chini ya – meza”, meaning “bottle – under – table”). Participants could then continue trying different positions until the correct one was found. Once an object was correctly placed, it was left on the corresponding box, and participants could move on to the next object. Every five minutes, Pepper assessed participants’ emotional states by asking them to rate how strongly they were experiencing enjoyment, boredom, and frustration using a five-point scale ranging from “not at all” to “very strongly”. These ratings were provided via a screen on the robot’s chest. Once all objects were correctly placed or after 30 min, the robot ended the session by thanking participants and asking them to leave the objects in place. The participants then returned to the experimenter’s station, located behind movable walls, where they completed the post-test.

### Experimental manipulation

The study incorporated a between-groups design with four experimental conditions, varying in the robots’ behavior. Between conditions, the amount of guidance provided alongside the feedback on correctness was manipulated. In condition 1, the robot provided simple guidance, indicating only whether the placement was correct or incorrect without further hints. Condition 2 involved enhanced guidance, where the robot not only provided feedback on correctness but also always additionally provided a hint after an incorrect placement. In condition 3, the robot adapted its level of guidance based on current task performance and on-task enjoyment of the participant, by adjusting the amount of guidance between simple guidance (0%) and enhanced guidance (100%) from experimental block to experimental block. Each emotion assessment, which occurred every 5 min, determined the transition to the next experimental block and therefore triggered a reevaluation of the current guidance level, which could either lead to a change or maintenance of the existing level for the next block. In condition 4, the robot both adapted the level of guidance and personalized its feedback based on the learner’s progress, adapting the hints to the specific mistakes made.

**Fig. 2:**
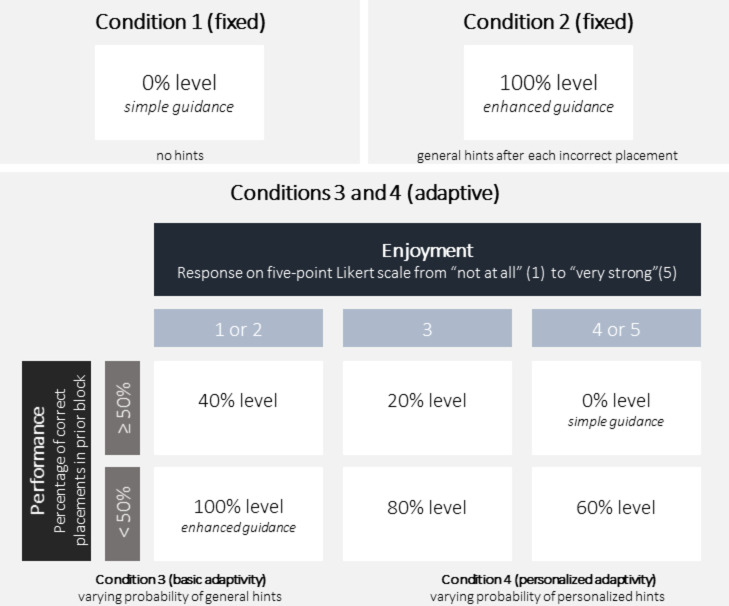
Illustration of the different conditions. The four conditions varied in the level and adaptivity of guidance provided by the robot. In condition 1 (simple guidance; fixed), the robot provided no hints beyond basic correctness feedback (0% guidance level). In condition 2 (enhanced guidance; fixed), the robot provided general hints after every incorrect action (100% guidance level) without adapting to the participant’s performance or emotions. In contrast, the adaptive robot in conditions 3 and 4 used a predefined matrix to determine the level of guidance based on participants’ performance (percentage of correct placements per block) and on-task enjoyment (response on a five-point Likert scale). Every five minutes, participants reported their emotions, and the adaptive robot used this information – along with performance data – to reevaluate which guidance level to follow, always starting at the 0% level (simple guidance). Higher performance led to fewer hints, while lower enjoyment triggered more frequent hints. Hints were given probabilistically based on the selected guidance level, and when given, they were randomly chosen from a predefined list. Conditions 3 (basic adaptivity) and 4 (personalized adaptivity) followed the same adaptive structure but different hints were used. While condition 3 provided general hints, condition 4 tailored hints to participants’ specific mistakes and individual learning progress.

In the adaptive conditions 3 and 4, the adaptive robot evaluated the participant’s task performance and emotional state every five minutes to determine the most suitable level of guidance. Based on this evaluation, the robot adjusted its behavior by providing hints at six possible levels (0%, 20%, 40%, 60%, 80%, or 100%). This decision was based on a predefined matrix considering participants’ performance in the last experimental block and the latest on-task enjoyment rating (see Fig. 2 for a visualization). The matrix was developed based on a hint delivery strategy proposed in prior research^42^, which also used emotional experience and task performance to determine the appropriate level of adaptive support. Accordingly, participants performing well – defined as at least half of the placements in the last experimental block being correct – received fewer hints, while lower enjoyment levels resulted in more frequent hints. Additionally, if a participant continuously demonstrated high task performance (≥ 50% of placements were correct) but decreased in enjoyment compared to the previous emotion assessment, the robot also delivered a motivational message alongside the regular hint. The robot varied the amount of guidance by providing hints based on a probability level. For example, if it decided to provide hints at the 20% level for an experimental block, after each mistake made by the participant in that block, the robot evaluated whether to give a hint, and a hint was given with a probability of 20% – resulting in fewer hints compared to for example the 80% guidance level. When a hint was provided, it was randomly selected from a list of predefined hints.

The hints provided by the robot were (meta-)cognitive hints, designed to encourage participants to reflect on their actions and strategies without providing direct solutions to the task. These hints were grounded in Zimmerman’s model of SRL^43^ and adapted from Nückles et al.^44^ to fit the specific task in this study. They covered a range of strategies to promote deeper engagement with the task, including cognitive organization and elaboration, as well as meta-cognitive monitoring and self-regulation. For example, hints like *“How could you break the sentences into smaller parts to make it easier?*” were aimed at helping participants organize their thoughts and understand the task, while others like “*What made you choose this position? Try to reflect on your steps*”, encouraged deeper reflection and regulation. In the personalized adaptivity condition, the hints were further tailored to specific types of mistakes made by the participants. For instance, when a participant made a mistake with a target location word they had encountered before and should have already known, the robot might offer a hint like *“How could you break the sentences into smaller parts to make it easier? In this case*,* for example*,* especially the last word could have helped you*”. Similarly, if a participant placed an object incorrectly and should have known the position was wrong, the robot could respond with a hint such as “What made you choose this position for *object*? Try to reflect on your steps – *you could have known it wasn’t correct*”. All hints were carefully adapted with feedback from trained educators to ensure they closely reflect how a human teacher would provide guidance, making the interactions as realistic and aligned with human teaching principles as possible.

### Measures

#### Task performance

Task performance was measured as the proportion of correctly placed objects relative to the total number of placements during the complete interaction. Each participant was tasked with placing 12 objects, and the number of correct placements was divided by the total number of attempts, yielding a task performance score between 0 and 1. In this sample, task performance ranged from a minimum of 0.05 to a maximum of 0.75, with a mean score of 0.35 (*SD* = 0.13), indicating that, on average, around one-third of each participant’s placements were correct.

#### Cognitive learning

Cognitive learning was assessed using a vocabulary test administered after the learning session. Participants were asked to translate ten Swahili words used during the task into German, measuring how many words they could remember beyond the interaction. The test consisted of audio recordings of the Pepper robot speaking each of the ten words at a time. The tested words included three objects, three prepositions, and four target locations. Each word was presented as an individual audio recording, and participants were allowed to play each audio clip twice. They could leave answers blank if they did not remember the word.

#### On-task emotions

Participants self-reported their emotional experience during the task based on control-value theory^18^. Specifically, they rated one positive (enjoyment), one negative (frustration), and one neutral activity emotion (boredom), using a short version of the epistemically-related emotion scale^45^. Participants rated the current strength of each emotion on a five-point Likert scale, ranging from “not at all” to “very strong”. These assessments were made every five minutes using the robot’s integrated touch screen, ensuring a brief and efficient assessment that minimally interrupted the learning process.

#### Self-regulated learning (SRL) behavior

SRL behavior was assessed through video recordings of participants during the task. These video recordings were analyzed using a modified version of Whitebread and Pino-Pasternak’s coding framework, designed to assess SRL in social and naturalistic contexts via video observation^46^. The framework evaluates SRL behavior in four metacognitive subdimensions: planning, monitoring, control/regulation, and reflection/evaluation. The category system was adopted in its entirety, but the behavioral examples were adapted to fit the experimental context and adult sample of this study. Subcategories related to peer interaction (e.g., correcting others’ performance) were excluded as no other learners were involved in the study. Prior to data collection, example behaviors were defined based on the original coding scheme^46^ and expectations of potential behaviors. After data collection, examples were further refined based on observations, and two raters evaluated whether behaviors fit the respective categories in accordance with the definitions of the original authors.

Planning was defined as “any verbalization or behavior related to the selection of procedures, necessary for performing the task”^47^. In this study, planning involved behaviors such as participants actively choosing the next object to place with a clear strategy, or counting the number of objects before beginning the task. Monitoring referred to “any verbalization or behavior related to the ongoing on-task assessment of the quality of task performance and the degree to which performance is progressing towards a desired goal”^47^. In this context, monitoring behaviors included participants checking how well they had performed so far, assessing previous placements, or recognizing when they made a mistake. Control/regulation was defined as “any verbalization or behavior related to a change in the way a task had been conducted, as a result of cognitive monitoring”^47^. In our study, this included participants adapting their task approach, such as putting an object back if they realized they were unsure of its placement or strategically linking and transferring the information provided by the robot to adjust their decisions. Finally, reflection/evaluation was defined as “any verbalization or behavior related to reviewing task performance and evaluating the quality of performance”^47^. This included behaviors reviewing overall performance such as participants reflecting on whether they were performing well or poorly, or tracking their progress by looking at their watch or counting how many objects are still left.

Video data were coded by two independent raters using the adapted coding framework and Mangold Interact software^48^. In accordance with the original coding system, relevant events were marked if the corresponding behaviors occurred. For analyses, the total number of occurrences of each of the four subdimensions of SRL was calculated for each five-minute experimental block. To ensure reliability, 25% of data were coded by both raters, while the rest was split equally between them. Inter-rater reliability (ICC) was calculated using the ICC3 statistic^49^ in the R psych package^50^. The total ICC score across all subdimensions was 0.90, with category-specific ICCs of 0.80 for planning, 0.83 for monitoring, 0.85 for control/regulation, and 0.76 for reflection/evaluation, indicating high reliability^49^.

#### Additional measures

Age was self-reported in an open text format, allowing participants to enter full numbers only. General cognitive ability was assessed using the short version of the Hagen Matrices Test (HMT-S), a tool designed to measure intelligent reasoning through pattern recognition and problem-solving involving matrices^51^. The HMT-S is a concise, six-item test that provides an efficient measure of cognitive ability, taking only a few minutes to complete. Its reliability and validity have been demonstrated, particularly in relation to academic performance and other intelligence measures^51^.

### Statistical analysis

To investigate the effects of adaptive teaching behavior of a social robot on students’ learning outcomes, emotional experiences, and SRL, three path models were constructed using Mplus Version 8.10^52^. The robust maximum likelihood (MLR) estimator was employed to account for non-normality. Each model assessed the relations of experimental conditions, cognitive ability, and age, with task performance and cognitive learning as outcome variables, but varied in the included mediators. The conditions were included as binary variables, with each type of guidance (simple guidance in condition 1, enhanced guidance in condition 2, and personalized adaptive guidance in condition 4) compared against the baseline adaptive guidance (condition 3). The first model (Model 1_DIRECT_) examined the direct effects of the conditions on the outcome variables cognitive learning and task performance while controlling for cognitive ability and age. The second model (Model 2_EMOTION_) utilized a two-level structure (specified as TYPE = TWOLEVEL in MPlus) to investigate how on-task emotions, repeatedly measured during the task, were influenced by the experimental block and the number of hints provided (within-level), and whether the experimental conditions indirectly affected both learning outcomes through on-task emotions (between-level). The third model (Model 3_SRL_) also employed a two-level approach but focused on SRL behaviors. More specifically, in this model, experimental blocks and the number of hints provided were regressed on the four SRL behavior subdimensions (within-level), and SRL behaviors were included as mediators of the relation between conditions and learning outcomes, examining indirect effects (between-level).

To evaluate the model fit, we applied established criteria as recommended by Tanaka^53^. A model was considered to have an acceptable fit if the Tucker-Lewis index (TLI) and comparative fit index (CFI) were ≥ 0.95, the root mean square error of approximation (RMSEA) was ≤ 0.06, and the standardized root mean residual (SRMR) was ≤ 0.10 for both within- and between-level effects^54^.

Due to the absence of video recordings, which led to missing SRL data, one participant was excluded from Model 3_SRL_, reducing the sample size to 119. However, this participant was included in the other two models. Additionally, non-existing cases, such as data for blocks participants did not complete because they ended the task earlier, were excluded. Aside from these instances, no other missing data were present across the model variables.

Based on the results, seven additional multilevel path models were calculated as part of post-hoc analyses, each including only one mediator (either one on-task emotion or one SRL behavior). These extra analyses aimed to detect potential indirect effects of the adaptive teaching behavior on learning outcomes that may not have been observable in the full models due to shared variance or high correlations between mediators. A detailed overview of the results from these post hoc analyses is provided in the Supplementary Information.

## Results

### Descriptive statistics and correlations

Table 1 provides descriptive statistics of study variables for the total sample and across the four experimental conditions. Pearson correlation coefficients are presented in Table 2 and reveal that the outcome variables, task performance and cognitive learning, were significantly and positively associated with each other. Boredom and frustration were both significantly negatively and enjoyment was positively and significantly correlated with task performance. Control/regulation was significantly and positively correlated with task performance and cognitive learning. Reflection/evaluation was significantly positively related to cognitive learning.

**Table 1:** Descriptive statistics.

	Total sample		Condition 1		Condition 2		Condition 3		Condition 4	
	*M*	*SD*	*M*	*SD*	*M*	*SD*	*M*	*SD*	*M*	*SD*
Age	30.25	10.06	32.40	9.49	28.30	10.02	29.97	9.97	30.33	10.79
Cognitive ability	4.08	1.37	3.67	1.56	4.40	1.13	4.07	1.23	4.17	1.46
Task performance	0.35	0.13	0.35	0.14	0.33	0.13	0.34	0.12	0.37	0.12
Cognitive learning	2.53	1.72	2.37	1.77	2.47	1.80	2.27	1.66	3.00	1.64
On-task enjoyment^a^	3.38	0.78	3.25	0.65	3.75	0.66	3.30	0.96	3.24	0.75
On-task boredom^a^	1.41	0.60	1.48	0.68	1.20	0.41	1.34	0.59	1.61	0.62
On-task frustration^a^	2.22	0.83	2.08	0.86	2.13	0.78	2.20	0.90	2.49	0.77
Planning^a^	0.17	0.45	0.12	0.24	0.11	0.22	0.28	0.79	0.16	0.28
Monitoring^a^	4.50	1.65	4.65	1.61	4.70	1.86	4.45	1.56	4.21	1.59
Control/regulation^a^	3.72	1.73	3.55	1.65	3.53	1.82	4.01	1.67	3.78	1.82
Reflection/evaluation^a^	0.32	0.34	0.27	0.43	0.29	0.27	0.27	0.23	0.46	0.37
Number of hints^a^	1.89	1.66	0.00	0.00	3.48	1.42	2.35	1.27	1.72	1.06

Descriptive statistics for all study variables are presented for the total sample (*N* = 120) and separately for the four conditions (condition 1: simple guidance (*n* = 30), condition 2: enhanced guidance (*n* = 30), condition 3: basic adaptive guidance (*n* = 30), and condition 4: personalized adaptive guidance (*n* = 30)). Variables marked with ^a^ represent level 1 (L1) variables, which were aggregated across all experimental blocks (mean values for each participant). *M* represents the mean, and *SD* represents the standard deviation.

**Table 2:** Bivariate correlations.

		1	2	3	4	5	6	7	8	9	10	11
1.	Age	–										
2.	Cognitive ability	− 0.28**	–									
3.	Task performance	− 0.28**	0.32**	–								
4.	Cognitive learning	− 0.28**	0.27**	0.47**	–							
5.	On-task enjoyment^a^	− 0.09	0.15	0.22*	0.01	–						
6.	On-task boredom^a^	0.14	− 0.25**	− 0.25**	− 0.01	− 0.53**	–					
7.	On-task frustration^a^	0.02	− 0.17	− 0.26**	0.01	− 0.54**	0.44**	–				
8.	Planning^a^	− 0.16	0.01	− 0.03	− 0.03	0.06	− 0.03	0.04	–			
9.	Monitoring^a^	− 0.20*	− 0.00	− 0.10	0.01	0.21*	− 0.16	0.05	− 0.01	–		
10.	Control/regulation^a^	− 0.35**	0.26**	0.65**	0.53**	0.22*	− 0.22*	− 0.13	0.13	− 0.02	–	
11.	Reflection/evaluation^a^	0.14	− 0.14	0.04	0.22*	− 0.05	0.01	0.15	− 0.08	0.16	0.12	–

Bivariate correlations (Pearson’s *r*) for study variables across all conditions. Correlations with * were significant at *p* < .05, and ** indicate significance at *p* < .01. Variables marked with ^a^ represent level 1 (L1) variables, which were aggregated across all experimental blocks (mean values for each participant).

### Direct effects of guidance level on task performance and cognitive learning (H1)

**Fig. 3:**
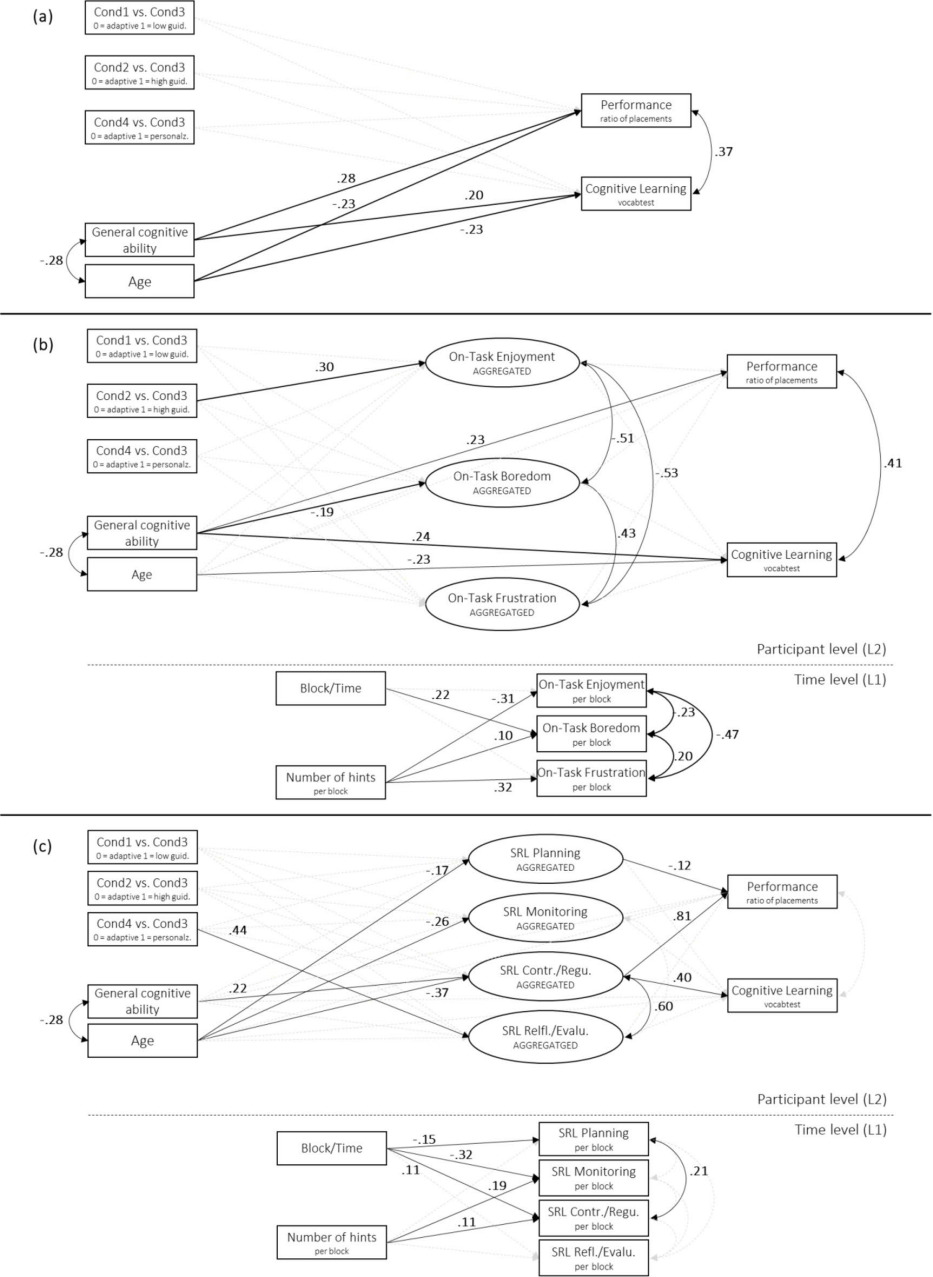
Visualization of all three path models including standardized coefficients. Solid, bold lines represent statistically significant standardized coefficients (*p* < .05), while dashed lines indicate nonsignificant relations between constructs. **(a)** Illustration of Model 1_DIRECT_, **(b)** Illustration of multilevel Model 2_EMOTION_, and **(c)** Illustration of multilevel Model 3_SRL_.

The effects of adaptive guidance (condition 3) on both task performance and cognitive learning were tested in comparison to all other three conditions (H1). Participants were assigned to one of four conditions: (1) simple guidance, where the robot provided feedback only on correctness, (2) enhanced guidance, where additional hints were given after incorrect placements, (3) adaptive guidance, where the robot adjusted the number of hints based on performance and enjoyment, and (4) personalized adaptive guidance, which further tailored hints to individual mistakes and progress. The path model, tested in MPlus with a single-level structure (TYPE = GENERAL) to test our hypothesis, had a good fit to the data (Model 1_DIRECT_: *χ²* = 5.88, df = 6, *p* = .437, RMSEA < 0.001, CFI = 1.00, TLI = 1.00, SRMR = 0.061). The results of the model, including all standardized coefficients, confidence intervals, and *p*-values, are reported in Table 3 and visualized in Fig. 3a. No significant direct effects of adaptive guidance (condition 3) in comparison to the simple guidance (condition 1) or enhanced guidance (condition 2) on both task performance and cognitive learning were shown. Further, adaptive guidance (condition 3) did not show significant direct effects on these outcomes in comparison to personalized adaptive guidance (condition 4). Cognitive ability was significantly and positively associated with cognitive learning and task performance, while age was negatively related to both learning outcomes.

**Table 3:** Standardized results of model 1_DIRECT_.

	ß	SE	95%CI	*p*
Cognitive learning				
Condition 1 vs. 3	0.08	0.10	[-0.12, 0.27]	0.437
Condition 2 vs. 3	0.01	0.11	[-0.20, 0.22]	0.911
Condition 4 vs. 3	0.18	0.10	[-0.01, 0.37]	0.057
Cognitive ability	0.20	0.10	[0.02, 0.39]	0.031
Age	-0.23	0.08	[-0.39, -0.08]	0.004
Task performance				
Condition 1 vs. 3	0.11	0.10	[-0.09, 0.30]	0.302
Condition 2 vs. 3	-0.08	0.09	[-0.26, 0.10]	0.396
Condition 4 vs. 3	0.11	0.09	[-0.07, 0.29]	0.243
Cognitive ability	0.28	0.07	[0.14, 0.42]	0.000
Age	-0.23	0.11	[-0.44, -0.01]	0.038

Standardized results of Model 1_DIRECT_, assessing direct effects of the experimental conditions on learning outcomes (H1). *ß* represents standardized coefficients, *SE* is the standard error, *95%CI* indicates the 95% confidence intervals, and p represents the *p*-value.

### Emotional experience as a mediator of task performance and cognitive learning (H2)

In a second model, the relations between adaptive guidance and the on-task emotions enjoyment, boredom, and frustration were examined (H2a), and we tested whether these emotions might mediate the relation between guidance levels and learning outcomes (H2b). To capture both time-level (within) and participant-level (between) effects, a multilevel path model with a two-level structure was specified in MPlus (TYPE = TWOLEVEL), and showed a good fit (Model 2_EMOTION_: *χ²* = 16.44, df = 12, *p* = .172, RMSEA = 0.026, CFI = 0.99, TLI = 0.94, SRMR_WITHIN_ = 0.003, SRMR_BETWEEN_ = 0.052). The results, including standardized coefficients, confidence intervals, and *p*-values, are reported in Table 4 and visualized in Fig. 3b. At the between-level, no significant indirect effects of conditions through emotional experience on cognitive learning and task performance were shown. However, enhanced guidance (condition 2) was significantly and positively related to on-task enjoyment, indicating higher enjoyment in the enhanced guidance condition compared to the adaptive guidance condition. Cognitive ability was significantly and positively associated with cognitive learning and task performance, while age was negatively related to cognitive learning. On the within-level, the number of hints per block were negatively related to on-task enjoyment and positively related to on-task boredom and on-task frustration. On-task boredom was significantly and positively correlated with the experimental block, indicating it increased over time.

**Table 4:** Standardized results of model 2_EMOTION_.

		ß	SE	95%CI	*p*			ß	SE	95%CI	*p*
**Between-level (L2)**						**Within-level (L1)**					
On-task enjoyment						On-task enjoyment					
	Condition 1 vs. 3	-0.13	0.13	[-0.38, 0.12]	0.314		Experimental block	0.06	0.07	[-0.08, 0.21]	0.385
	Condition 2 vs. 3	0.30	0.12	[0.06, 0.54]	0.013		Number of hints	-0.31	0.07	[-0.44, -0.18]	0.000
	Condition 4 vs. 3	-0.08	0.13	[-0.33, 0.16]	0.509	On-task boredom					
	Cognitive ability	0.11	0.08	[-0.05, 0.28]	0.180		Experimental block	0.22	0.07	[0.09, 0.35]	0.001
	Age	-0.01	0.10	[-0.21, 0.20]	0.943		Number of hints	0.10	0.05	[0.00, 0.20]	0.041
On-task boredom						On-task frustration					
	Condition 1 vs. 3	0.13	0.13	[-0.12, 0.38]	0.301		Experimental block	0.03	0.09	[-0.14, 0.20]	0.736
	Condition 2 vs. 3	0.13	0.10	[-0.33, 0.08]	0.215		Number of hints	0.32	0.07	[0.19, 0.45]	0.000
	Condition 4 vs. 3	0.23	0.12	[-0.00, 0.47]	0.054	**Indirect effects (L2)**					
	Cognitive ability	-0.19	0.09	[-0.38, -0.01]	0.039	Cognitive learning					
	Age	0.04	0.09	[-0.14, 0.23]	0.649		Condition 1 vs. 3 → On-task enjoyment	0.00	0.02	[-0.03, 0.03]	0.999
On-task frustration							Condition 1 vs. 3 ﻿→ On-task boredom	0.01	0.02	[-0.03, 0.05]	0.652
	Condition 1 vs. 3	0.05	0.13	[-0.20, 0.30]	0.701		Condition 1 vs. 3 ﻿→ On-task frustration	0.00	0.01	[-0.02, 0.03]	0.753
	Condition 2 vs. 3	-0.08	0.13	[-0.32, 0.16]	0.521		Condition 2 vs. 3 ﻿→ On-task enjoyment	0.00	0.04	[-0.08, 0.08]	0.999
	Condition 4 vs. 3	0.22	0.12	[-0.01, 0.15]	0.056		Condition 2 vs. 3 ﻿→ On-task boredom	-0.01	0.02	[-0.04, 0.02]	0.566
	Cognitive ability	-0.20	0.11	[-0.41, 0.14]	0.060		Condition 2 vs. 3 ﻿→ On-task frustration	-0.01	0.01	[-0.04, 0.02]	0.650
	Age	-0.06	0.11	[-0.26, 0.33]	0.564		Condition 4 vs. 3 ﻿→ On-task enjoyment	0.00	0.01	[-0.02, 0.02]	0.999
Cognitive learning							Condition 4 vs. 3 ﻿→ On-task boredom	0.02	0.03	[-0.05, 0.08]	0.610
	On-task enjoyment	0.00	0.13	[-0.25, 0.32]	0.999		Condition 4 vs. 3 ﻿→ On-task frustration	0.02	0.03	[-0.04, 0.08]	0.542
	On-task boredom	0.07	0.13	[-0.16, 0.34]	0.567	Task performance					
	On-task frustration	0.09	0.13	[0.06, 0.41]	0.488		Condition 1 vs. 3 ﻿→ On-task enjoyment	-0.00	0.02	[-0.04, 0.04]	0.916
	Cognitive ability	0.24	0.09	[-0.38, 0.25]	0.010		Condition 1 vs. 3 ﻿→ On-task boredom	-0.01	0.02	[-0.04, 0.03]	0.746
	Age	-0.23	0.08	[-0.38, 0.21]	0.004		Condition 1 vs. 3 ﻿→ On-task frustration	-0.00	0.01	[-0.03, 0.02]	0.726
Task performance							Condition 2 vs. 3 ﻿→ On-task enjoyment	0.01	0.05	[-0.08, 0.09]	0.915
	On-task enjoyment	0.02	0.15	[-0.30, 0.15]	0.916		Condition 2 vs. 3 ﻿→ On-task boredom	0.01	0.02	[-0.03, 0.04]	0.748
	On-task boredom	-0.04	0.13	[-0.33, 0.38]	0.747		Condition 2 vs. 3 ﻿→ On-task frustration	0.01	0.01	[-0.02, 0.03]	0.547
	On-task frustration	-0.09	0.12	[0.09, 0.38]	0.475		Condition 4 vs. 3 ﻿→ On-task enjoyment	-0.00	0.01	[-0.03, 0.02]	0.915
	Cognitive ability	0.23	0.08	[-0.37, -0.36]	0.002		Condition 4 vs. 3 ﻿→ On-task boredom	-0.01	0.03	[-0.07, 0.05]	0.746
	Age	-0.21	0.12	[-0.35, -0.64]	0.069		Condition 4 vs. 3 ﻿→ On-task frustration	-0.02	0.03	[-0.07, 0.03]	0.482

Standardized results of multilevel Model 2_EMOTION_, assessing indirect effects of experimental conditions on learning outcomes through on-task emotions (H2). *ß* represents standardized coefficients, *SE* is the standard error, *95%CI* indicates the 95% confidence intervals, and *p* represents the *p*-value.

### Self-regulated learning as a mediator of task performance and cognitive learning (H3)

In a third model, we tested whether SRL behaviors – more specifically planning, monitoring, control/regulation, and reflection/evaluation – were affected by adaptive guidance (H3a) and whether they would mediate the relation between guidance levels and learning outcomes (H3b). The multilevel path model showed a good fit (Model 3_SRL_: *χ²* = 20.36, df = 16, *p* = .204, RMSEA = 0.022, CFI = 0.99, TLI = 0.94, SRMR_WITHIN_ = 0.009, SRMR_BETWEEN_ = 0.056). The results are visualized in Fig. 3c and all standardized coefficients, confidence intervals, and *p*-values are reported in Table 5. At the between-level, no significant indirect effects of adaptive guidance through SRL behaviors on cognitive learning and task performance were observed. However, control/regulation was positively associated with task performance as well as cognitive learning. Further, higher planning was significantly related to lower task performance. Personalized adaptive guidance (condition 4) was significantly and positively related to reflection/evaluation as compared to basic adaptive guidance (condition 3). Post-hoc analyses, assessing each mediator individually in separate models (see Supplementary Information SI1 for details on post-hoc analyses), revealed a significant positive indirect effect of personalized adaptive guidance on both learning outcomes through reflection/evaluation. However, in the main analyses, reflection/evaluation was not significantly related to task performance or cognitive learning. Age was significantly and negatively associated with the SRL dimensions planning, monitoring, and control/regulation. Additionally, cognitive ability was significantly and positively correlated with control/evaluation. At the within level, the number of hints per block were significantly and positively related to monitoring and control/regulation. The experimental block was positively related to control/regulation, while it was negatively associated with planning and monitoring. No significant effect of hints or the experimental block was observed on reflection/evaluation.

**Table 5:** Standardized results of Model3_SRL_.

		ß	SE	95%CI	*p*			ß	SE	95%CI	*p*
**Between-level (L2)**						**Within-level (L1)**					
Planning						Planning					
	Condition 1 vs. 3	-0.16	0.08	[-0.33, 0.00]	0.056		Experimental block	-0.15	0.05	[-0.25, -0.06]	0.002
	Condition 2 vs. 3	-0.19	0.10	[-0.38, 0.01]	0.066		Number of hints	0.02	0.05	[-0.08, 0.13]	0.690
	Condition 4 vs. 3	-0.12	0.11	[-0.33, 0.09]	0.249	Monitoring					
	Cognitive ability	-0.06	0.05	[-0.15, 0.03]	0.201		Experimental block	-0.32	0.04	[-0.40, -0.24]	0.000
	Age	-0.17	0.05	[-0.27, -0.08]	0.000		Number of hints	0.19	0.05	[0.08, 0.29]	0.000
Monitoring						Control/regulation					
	Condition 1 vs. 3	0.17	0.16	[-0.14, 0.47]	0.280		Experimental block	0.11	0.05	[0.02, 0.21]	0.019
	Condition 2 vs. 3	0.01	0.14	[-0.27, 0.28]	0.971		Number of hints	0.11	0.05	[0.01, 0.20]	0.029
	Condition 4 vs. 3	-0.05	0.13	[-0.30, 0.21]	0.730	Reflection/evaluation					
	Cognitive ability	-0.11	0.11	[-0.33, 0.11]	0.335		Experimental block	0.08	0.05	[-0.01, 0.17]	0.086
	Age	-0.26	0.12	[-0.50, -0.01]	0.038		Number of hints	0.07	0.05	[-0.03, 0.17]	0.170
Control/regulation						**Indirect effects (L2)**					
	Condition 1 vs. 3	0.09	0.11	[-0.12, 0.31]	0.404	Cognitive learning					
	Condition 2 vs. 3	-0.16	0.12	[-0.40, 0.08]	0.183		Condition 1 vs. 3 ﻿→ Planning	0.01	0.01	[-0.02, 0.04]	0.376
	Condition 4 vs. 3	0.02	0.17	[-0.31, 0.35]	0.901		Condition 1 vs. 3 ﻿→ Monitoring	-0.03	0.04	[-0.10, 0.04]	0.466
	Cognitive ability	0.22	0.09	[0.05, 0.40]	0.014		Condition 1 vs. 3 ﻿→ Control/regulation	0.04	0.05	[-0.06, 0.13]	0.453
	Age	-0.37	0.10	[-0.57, -0.17]	0.000		Condition 1 vs. 3 ﻿→ Reflection/evaluation	0.04	0.10	[-0.15, 0.23]	0.675
Reflection/evaluation							Condition 2 vs. 3 ﻿→ Planning	0.01	0.02	[-0.02, 0.05]	0.391
	Condition 1 vs. 3	0.11	0.22	[-0.31, 0.53]	0.609		Condition 2 vs. 3 ﻿→ Monitoring	-0.00	0.02	[-0.04, 0.04]	0.971
	Condition 2 vs. 3	0.08	0.15	[-0.21, 0.38]	0.587		Condition 2 vs. 3 ﻿→ Control/regulation	-0.07	0.05	[-0.16, 0.03]	0.187
	Cond 4 vs. 3	0.44	0.21	[0.03, 0.84]	0.034		Condition 2 vs. 3 ﻿→ Reflection/evaluation	0.03	0.06	[-0.09, 0.15]	0.630
	Cognitive ability	-0.17	0.18	[-0.52, 0.18]	0.344		Condition 4 vs. 3 ﻿→ Planning	0.01	0.01	[-0.02, 0.04]	0.484
	Age	0.10	0.13	[-0.15, 0.34]	0.438		Condition 4 vs. 3 ﻿→ Monitoring	0.01	0.02	[-0.03, 0.05]	0.727
Cognitive learning							Condition 4 vs. 3 ﻿→ Control/regulation	0.01	0.07	[-0.13, 0.14]	0.904
	Planning	-0.08	0.06	[-0.20, 0.04]	0.216		Condition 4 vs. 3 ﻿→ Reflection/evaluation	0.16	0.12	[-0.08, 0.40]	0.198
	Monitoring	-0.15	0.18	[-0.50, 0.20]	0.388	Task performance					
	Control/regulation	0.40	0.20	[0.01, 0.79]	0.045		Condition 1 vs. 3 ﻿→ Planning	0.02	0.02	[-0.01, 0.05]	0.232
	Reflection/evaluation	0.36	0.23	[-0.10, 0.82]	0.122		Condition 1 vs. 3 ﻿→ Monitoring	-0.03	0.04	[-0.11, 0.05]	0.439
	Cognitive ability	0.16	0.11	[-0.06, 0.37]	0.152		Condition 1 vs. 3 ﻿→ Control/regulation	0.07	0.10	[-0.11, 0.26]	0.437
	Age	-0.17	0.16	[-0.49, 0.14]	0.279		Condition 1 vs. 3 ﻿→ Reflection/evaluation	0.01	0.05	[-0.09, 0.10]	0.871
Task performance							Condition 2 vs. 3 ﻿→ Planning	0.02	0.02	[-0.01, 0.06]	0.207
	Planning	-0.12	0.05	[-0.21, -0.02]	0.022		Condition 2 vs. 3 ﻿→ Monitoring	-0.00	0.03	[-0.05, 0.05]	0.971
	Monitoring	-0.18	0.21	[-0.60, 0.24]	0.395		Condition 2 vs. 3 ﻿→ Control/regulation	-0.13	0.08	[-0.29, 0.03]	0.112
	Control/regulation	0.81	0.23	[0.35, 1.26]	0.001		Condition 2 vs. 3 ﻿→ Reflection/evaluation	0.01	0.03	[-0.06, 0.07]	0.856
	Reflection/evaluation	0.07	0.32	[-0.56, 0.71]	0.828		Condition 4 vs. 3 ﻿→ Planning	0.01	0.02	[-0.02, 0.05]	0.383
	Cognitive ability	0.08	0.11	[-0.13, 0.29]	0.431		Condition 4 vs. 3 ﻿→ Monitoring	0.01	0.03	[-0.04, 0.06]	0.743
	Age	0.00	0.19	[-0.36, 0.36]	0.999		Condition 4 vs. 3 ﻿→ Control/regulation	0.02	0.14	[-0.25, 0.29]	0.904
							Condition 4 vs. 3 ﻿→ Reflection/evaluation	0.03	0.15	[-0.26, 0.32]	0.838

Standardized results of multilevel Model 3_SRL_, assessing indirect effects of experimental conditions on learning outcomes through on-task emotions (H2). *ß* represents standardized coefficients, *SE* is the standard error, *95%CI* indicates the 95% confidence intervals, and *p* represents the *p*-value.

## Discussion

The present study examined the impact of adaptive teaching behavior in a social robot on students’ learning outcomes, emotional experiences, and SRL behaviors. Participants engaged in an interactive vocabulary learning task, with the social robot varying in the level of guidance between experimental conditions. In the control conditions, the robot provided a fixed level of guidance, either offering simple guidance with no hints (condition 1), or enhanced guidance with additional hints after each incorrect action (condition 2). In the adaptive conditions, the robot adjusted the level of hints based on the learner’s performance and emotional state (condition 3), or additionally personalized the hints based on the learner’s progress and specific mistakes (condition 4).

The key results were that adaptive teaching (condition 3) did not directly affect task performance or cognitive learning (H1) when compared to the non-adaptive control conditions. There were no significant indirect effects of adaptive teaching on learning outcomes mediated by emotional experiences (H2b) or SRL behaviors (H3b) in comparison to the non-adaptive conditions. Enhanced guidance (condition 2), however significantly increased on-task enjoyment compared to adaptive guidance (H2a). Our findings showed a significant positive effect of personalized adaptive guidance (condition 4) on reflection/evaluation compared to basic adaptive guidance (condition 3), with post-hoc analyses revealing a significant indirect effect on learning outcomes. These findings offer relevant insights into the ongoing research on adaptive social robots and their role in supporting student learning by proposing potential indirect effects via SRL behaviors.

Our main finding was that, contrary to our initial hypotheses, the adaptive teaching behavior of the robot did not have the expected direct positive effects on learning outcomes (H1), emotional experience (H2), or SRL behaviors (H3). While Vygotsky’s ZPD suggests that finding the right balance between too much and too little support is essential for effective learning^14^, and prior studies on human teachers suggest that adaptive guidance outperforms fixed levels of support^17,21,55^, these findings could not be replicated in this study. A potential explanation for why adaptive guidance did not improve outcomes may lie in the limitations of the adaptive model used in our study, which may not have been sufficiently dynamic or individualized. The robot’s adaptivity was guided by a predefined decision matrix, updating its guidance level only every five minutes based on performance and enjoyment ratings. While this approach was based on prior research^42^, it may have lacked the real-time responsiveness needed for effective adaptation. Participants’ expectations about interactions with robots may have contributed to this effect, as prior studies have shown that people apply human-to-human interaction scripts in human-robot interactions and may have perceived the robot’s fixed adaptation intervals and randomly selected hints as unnatural and inconsistent with their expectations^56^.

Surprisingly, adapting the amount of guidance even negatively impacted students’ on-task enjoyment, as participants in condition 2 (enhanced guidance) showed significantly more on-task enjoyment then those in the adaptive condition (condition 3). The fact that participants reported more enjoyment in the enhanced guidance condition, despite receiving the same hints as in the adaptive condition, suggests that consistent, predictable feedback from a social robot may foster a more enjoyable learning experience. In the enhanced guidance condition, participants received a hint after every incorrect action, which might have reduced uncertainty in the robot’s role and contributed to a more comfortable learning situation through continuous support. In contrast, in the adaptive condition, the number of hints varied based on current performance and emotional experience, which created more variability in the robot’s behavior. This variability may have been perceived as less engaging, even though it was intended to tailor guidance to individual needs which was hypothesized to positively influence learning emotions. Instead, these findings suggest that varying, adaptive behavior in social robots may not always yield positive effects in human-robot interactions, as also indicated in related research^57^. While adapting the level of guidance might be an effective approach for human teachers, transferring this concept taken from human social interactions to interactions involving social robots might not be as straightforward. When interacting with a robot, humans may expect a more consistent and predictable behavior, an essential factor contributing to perceived safety in such interactions, as people tend to feel more comfortable when a robot behaves in a stable, foreseeable manner^58^.

Another main finding of our study is that adaptive guidance of a social robot fosters self-regulation in learners. The results showed that participants receiving personalized adaptive guidance (condition 4) showed significantly more reflection and evaluation compared to those receiving basic adaptive guidance (condition 3). Post-hoc analyses further revealed that personalized feedback can indirectly improve both task performance and cognitive learning by encouraging learners to reflect on and evaluate their performance more intensively. This positive impact of personalized adaptivity on reflection and evaluation suggests that when learners receive feedback that is specific to their mistakes and progress, they are more likely to reflect on their learning progress and evaluate what adjustments might be needed, ultimately leading to improved learning outcomes. In contrast, more general feedback may not trigger the same level of self-regulation. These findings align with prior research suggesting that adaptivity in the form of personalized feedback enhances students’ learning outcomes and increases SRL behaviors^59–61^.

A third main finding of our study was that simply adjusting the frequency of non-personalized hints (basic adaptivity) did not result in increased SRL behaviors compared to no or consistent hint delivery. This may be because the non-personalized hints were not fully processed or even ignored, particularly since participants were not required to respond to or address the hints. Thus, when automated feedback lacks personalization, learners may disregard or overlook it. Prior research also highlights that SRL prompts do not always have the desired effect, as students sometimes ignore or feel restricted by them, emphasizing the importance of deeper process analyses to understand how students interact with and respond to such prompts^62,63^.

An interesting secondary finding was that the (meta-)cognitive hints provided by the robot in this study, across conditions, increased monitoring and control/regulation. However, the number of hints per block was negatively related to subsequent ratings of on-task enjoyment and positively associated with subsequent ratings of both on-task boredom and frustration. This negative effect of increased external regulation, through the more frequent delivery of hints, could be explained by control-value theory^18^. According to this theory, frequent external support might reduce the learner’s perceived control over the learning activity, leading to negative emotions such as frustration and boredom^18^. This highlights the challenge of balancing cognitive support with emotional engagement when designing adaptive technologies, as too much external guidance may negatively affect learners’ emotional experience, even if it enhances their SRL behaviors. Again, this challenge could be particularly relevant when modeling such (meta-)cognitive guidance in an adaptive social robot, as the variability in robot behavior may not align with learners’ expectations, further contributing to emotional disengagement.

Another secondary finding pertained to the covariates in the model. Age and general cognitive ability were important in predicting learning success, even more important than the guidance provided by the robot. Both age and cognitive ability significantly related to task performance and cognitive learning, with younger participants and those with higher cognitive ability performing better across both measures. Notably, age and cognitive ability were also negatively correlated, meaning that older participants tended to have lower cognitive ability scores. This relation might be explained through patterns of cognitive aging, which can impact memory, processing speed, and problem-solving skills^64^ – important factors for this study’s learning tasks. In contrast, younger participants, mostly university students, likely had more recent experience with similar cognitive tasks, which better prepared them for the challenges of the study. Additionally, older participants may have been more motivated by financial compensation, which could have reduced their overall engagement. Alternative explanations for the age-related findings include differences in technological familiarity, with older participants potentially being less accustomed to interacting with robots, and variations in task perception, as they may have found the task more challenging or unfamiliar. These factors, along with cognitive ability and motivation, highlight the need for adaptive instructional systems that consider individual learner characteristics to provide effective, personalized support.

The theoretical contribution of this study to current research is that we extend existing knowledge on learning processes and how learning outcomes can be fostered by adaptivity in human-robot interaction. We provide insights into the potential of adaptive social robots in educational settings, particularly regarding their role in students’ emotional experiences, SRL behaviors, and learning outcomes. The findings suggest that personalized adaptive guidance could be a key factor in enhancing reflection and evaluation, which could indirectly improve learning outcomes. However, our findings also show that variability in guidance, when delivered by a robot, may not always be perceived as supportive, even when it is adaptively tailored to the individual learner. To refine adaptive systems, future efforts should focus on improving feedback timing through more dynamic and real-time adjustments, potentially leveraging machine learning algorithms to enhance responsiveness. Additionally, personalization could be strengthened by tailoring hints to specific learner challenges, ensuring that support is both relevant and timely. Incorporating multimodal data, such as physiological responses or behavioral patterns, may further improve the system’s ability to adapt effectively to learners’ needs. Moreover, refining the way adaptive strategies are communicated to users can help set appropriate expectations and increase acceptance of variability in guidance. Finally, the strong influence of learner characteristics, such as age and cognitive ability, underscores the need to design adaptive systems that account for individual differences to provide effective and personalized support.

Although this study provides valuable insights into the role of a social robot’s adaptive guidance in an interactive learning scenario, several limitations must be acknowledged. The absence of direct effects of the guidance level on task performance and vocabulary test results challenges the assumption that adaptive guidance, as implemented in this study, leads to measurable improvements in learning outcomes. One possible explanation for the lack of significant results is that the form of adaptivity implemented may have been too simplistic and not sufficiently dynamic or responsive to individual learners’ needs, as it yields several limitations. First, the robot’s adaptive behavior adjusted the strategy only once every five minutes. This relatively slow adjustment process may not have provided the real-time responsiveness needed for effective adaptation, potentially reducing the expected benefits of adaptive guidance on learning outcomes. Second, although the robot adaptively decided whether to give more or fewer hints, the specific hints were chosen randomly from a list rather than being tailored to the participant’s particular challenges. For example, if a participant already understood the sentence structure of the robot’s input but struggled with recalling a certain word, the robot might still provide a hint explaining the sentence structure instead of focusing on the word recall issue. As a result, although the system dynamically regulated the quantity of hints, the fixed and non-individualized nature of the feedback may have diminished its overall effectiveness in addressing learners’ actual needs. This may explain why adaptive guidance did not lead to significant improvements in task performance or cognitive learning, as the hints might not have provided the targeted scaffolding necessary for deeper understanding. Although the personalized adaptive guidance (condition 4) was an improvement towards more individualized support and showed indirect effects on learning outcomes, there is still room to tailor the content of the hints more effectively. Third, the robot’s ability to adapt to participants’ emotions was limited to on-task enjoyment, excluding other potentially important emotional factors like frustration, which could have offered a more complete understanding of the learner’s experience. Research suggests that emotions beyond enjoyment, such as frustration and confusion, are essential in learning, as they can signal cognitive challenges and the need for additional motivational support^16^. Finally, the adaptivity concept did not account for additional relevant factors, such as learner characteristics like age and cognitive ability, which – as results indicate – were significant predictors of learning outcomes in this study. Cognitive aging effects, differences in technological familiarity, or variations in motivation may have influenced task engagement and, consequently, performance, potentially explaining why the effects of adaptive teaching on learning outcomes were not evident. A more effective adaptivity concept might involve adjusting guidance not only based on situational learning aspects but also on individual learner characteristics. These limitations suggest that, while adaptive systems show potential, the specific design and implementation of adaptive guidance – particularly in the context of social robots – require further refinement. A more dynamic, real-time adjustment process and more tailored feedback strategies may be necessary to achieve the desired learning improvements.

In addition to the limitations of the adaptivity concept, there are other factors to consider. The use of Swahili vocabulary, an unfamiliar language to participants, may have introduced a novelty effect, potentially boosting initial motivation and engagement, which could limit the generalizability of the findings. Furthermore, despite efforts to achieve a diverse sample by not restricting recruitment to university students or a certain age group, the sample was predominantly composed of university students, with limited representation from other demographic groups, a majority of native German speakers, and no inclusion of younger, school-aged learners. Also, self-reported emotion measures have limitations, such as reliance on participants’ awareness and honesty, potential biases, and delays in capturing real-time emotional states. While self-reports were used in this study for their reliability, as current real-time emotion detection systems remain prone to inaccuracies and inconsistencies, alternative approaches could provide more precise emotion tracking. Future studies may consider integrating physiological data, such as heart rate variability or electrodermal activity, to obtain continuous, objective indicators of emotional arousal^65^. Another approach could involve participant-initiated self-reporting, allowing individuals to log their emotions when they feel a change, rather than at predefined intervals. These alternative methods could improve the accuracy of emotion assessment in adaptive learning systems while maintaining feasibility and reliability in practical implementations. Despite these limitations, this study provides a solid foundation for understanding adaptive behavior in social interactions, a highly complex concept that requires breaking it down into manageable components to model effectively in robots.

Practical implications of our study are that we were able to show that adaptive social robots might have the potential to offer more personalized and effective support in learning settings, helping to address the diverse needs of learners. More concretely, we show that social robots that adapted the level of guidance and additionally personalized its feedback based on the learner’s progress and the specific mistakes made, are particularly promising when aiming to foster learners’ self-regulation in educational settings. Future research should explore the practical application of adaptive guidance in diverse educational contexts, such as real-world classroom with younger, school-aged learners, to assess its applicability across different environments. By further refining these systems and exploring their integration into everyday educational practices, adaptive social robots could become valuable tools for enhancing personalized learning experiences.

## Electronic supplementary material

Below is the link to the electronic supplementary material.

Supplementary Material 1

## Data Availability

The datasets used and analyzed during the current study are available from the corresponding author on reasonable request.
