# Supplementary material for: How adaptive social robots influence cognitive, emotional, and self-regulated learning
Source: Sci Rep. 2025 Feb 24;15:6581. doi: 10.1038/s41598-025-91236-0 (PMC11850589; doi:10.1038/s41598-025-91236-0)
Supplement: Supplementary file 1 — Supplementary Material 1 [file 41598_2025_91236_MOESM1_ESM.pdf]

Supplementary Information

How adaptive social robots influence cognitive, emotional, and self-regulated learning

Helene Ackermann[1,2,*], Anna L. Lange[2,3], Verena V. Hafner[2,3], and Rebecca Lazarides[1,2]

[1]Department of Educational Sciences, Universität Potsdam, Karl-Liebknecht-Straße 24/25, 14476 Potsdam, Germany

[2]Science of Intelligence, Research Cluster of Excellence, Marchstraße 23, 10587 Berlin, Germany

[3]Department of Computer Science, Humboldt-Universität zu Berlin, Unter den Linden 6, 10099 Berlin, Germany

*Corresponding author: helene.ackermann@uni-potsdam.de

Supplementary Information SI1

To further explore the effects of a social robot's adaptive teaching behavior on learning outcomes, a series of post-hoc analyses were conducted in addition to the three path models addressing the main research question. Those analyses aimed to assess the influence of each mediator individually, focusing on specific on-task emotions and self-regulated learning (SRL) behaviors. By examining each mediator separately, the goal was to identify any potential indirect effects that may have been overlooked in the main models, which considered multiple mediators simultaneously.

Methods

In the post-hoc analyses, we examined the role of each mediator individually to assess their potential indirect effects on learning outcomes. These mediators included the on-task emotions enjoyment (Model S1_{ENJOY}), boredom (Model S2_{BORED}), and frustration (Model S3_{FRUST}), as well as the SRL behaviors of planning (Model S4_{PLAN}), monitoring (Model S5_{MON}), control/regulation (Model $\text{S6}_{\text{CONT/RE}}$), and reflection/evaluation (Model $\text{S7}_{\text{REFL/EV}}$). Each mediator was analyzed in a separate path model, using the same multilevel modeling approach as in the main analyses. One participant was excluded from SRL models due to missing data ($n = 119$), but included in the emotion-based models ($n = 120$).

Results

On-task emotions

Tables S1, S2, and S3 present the results of Models $S1_{ENJOY}$, $S2_{BORED}$, and $S3_{FRUST}$, which assess the individual roles of on-task emotions (enjoyment, boredom, and frustration) as mediators of the relationship between adaptive guidance and learning outcomes. Visualizations of these models can be found in Figure S1. On the between-level, no significant indirect effects of experimental conditions through emotional experience on task performance and cognitive learning were observed. However, enhanced guidance (condition 2) was significantly and positively related to on-task enjoyment, indicating higher enjoyment compared to the adaptive guidance condition. Personalized adaptive guidance (condition 4) was significantly and positively related to on-task boredom. On the within-level, the number of hints per block was negatively related to on-task enjoyment and positively related to both on-task boredom and on-task frustration. Additionally, on-task boredom was significantly and positively correlated with the experimental block, indicating that boredom increased over time.

SRL behaviors

Tables S4, S5, S6, and S7 present the results of Models $S4_{PLAN}$, $S5_{MON}$, $S6_{CONT/RE}$, and $S7_{REFL/EV}$, which examine the roles of SRL behaviors (planning, monitoring, control/regulation, and reflection/evaluation) as individual mediators. These results are visualized in Figure S2. On the between-level, simple guidance (condition 1) negatively impacted planning as compared to the adaptive guidance condition, while no significant effects were observed for monitoring or control/regulation. Personalized adaptive guidance (condition 4) was positively related to reflection/evaluation. Control/regulation and reflection/evaluation were positively and significantly associated with task performance as well as cognitive learning, whereas planning and monitoring had no significant effects. Indirect effects of the conditions on cognitive learning and task performance were generally non-significant, except for a significant and positive indirect effect of personalized adaptive guidance (condition 4) on both cognitive learning and task performance through reflection/evaluation. On the within-level, planning and monitoring were negatively correlated with the experimental block, indicating these activities decreased over time. However, control/regulation was positively associated with the experimental block. The number of hints was positively related to monitoring.

Discussion

The post-hoc analyses provided additional insights that were not fully captured in the main analyses. Most notably, the post-hoc models revealed a significant positive indirect effect of personalized adaptive guidance (condition 4) on both cognitive learning and task performance through reflection/evaluation. This finding did not emerge in the main analyses, where reflection/evaluation was not significantly associated with learning outcomes. This could be explained by multicollinearity,

particularly the high correlation between reflection/evaluation and control/regulation, suggesting that control/regulation may have suppressed the effect in the model that included all SRL mediators simultaneously. Additionally, the post-hoc results highlighted that personalized adaptive guidance also significantly increased on-task boredom – another finding not observed in the main analyses. This suggests that while personalized guidance may enhance certain SRL behaviors, it may also lead to increased boredom. Furthermore, the post-hoc analyses revealed that simple guidance (condition 1) negatively impacted planning compared to adaptive guidance. This suggests that students receiving only simple guidance may have been less likely to engage in proactive planning, possibly due to the complete absence of metacognitive hints, which were provided in the adaptive guidance condition.

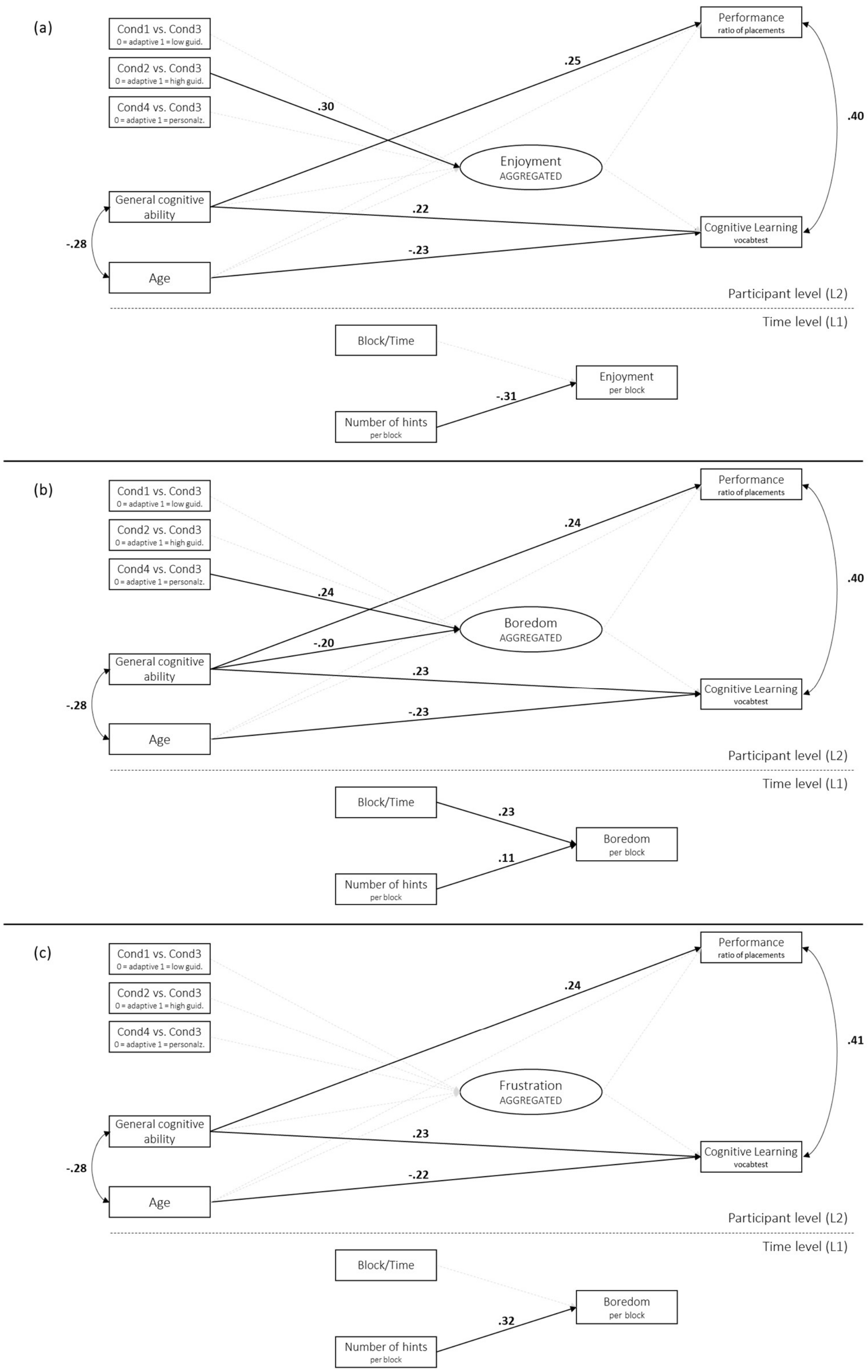

Supplementary Figure S1. Visualization of the three path models with on-task emotions as mediators, including standardized coefficients. Solid, bold lines represent statistically significant standardized coefficients ($p < .05$), while dashed lines indicate nonsignificant relations between constructs. **(a)** Illustration of multilevel Model $S1_{\text{ENJOY}}$, **(b)** Illustration of multilevel Model $S2_{\text{BORED}}$, and **(c)** Illustration of multilevel Model $S3_{\text{FRUST}}$.

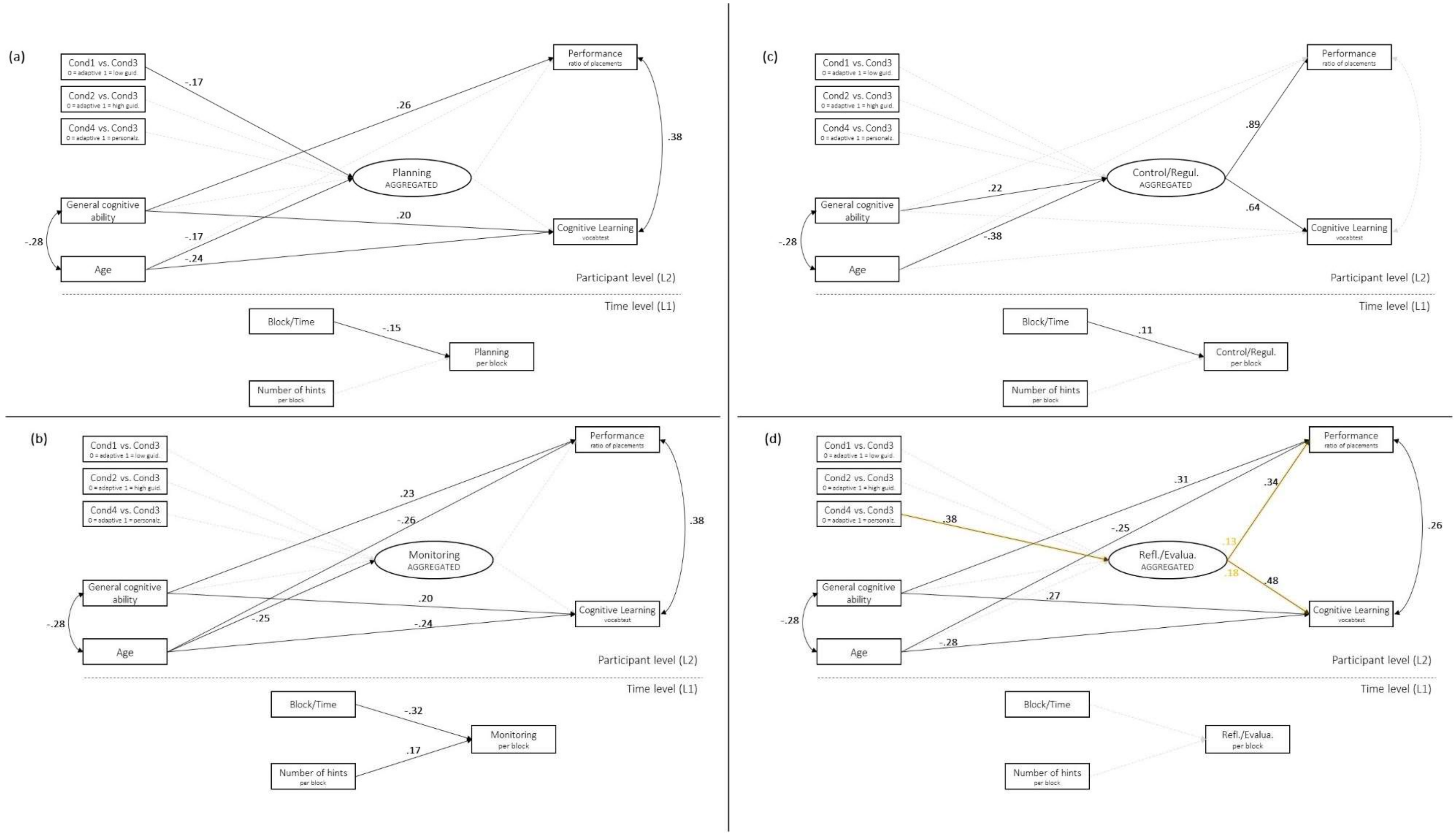

Supplementary Figure S2. Visualization of the four path models with SRL behaviors as mediators, including standardized coefficients. Solid, bold lines represent statistically significant standardized coefficients ($p < .05$), while dashed lines indicate nonsignificant relations between constructs. Yellow lines highlight significant indirect effects. **(a)** Illustration of multilevel Models S4$_{\text{PLAN}}$, **(b)** Illustration of multilevel Model S5$_{\text{MON}}$, **(c)** Illustration of multilevel Model S6$_{\text{CONT/RE}}$, and **(d)** Illustration of multilevel Model S7$_{\text{REFL/EV}}$.

Supplementary Table S1. Standardized results of Model $S1_{ENJOY}$.

	ß	*SE*	*95%CI*	*p*		*ß*	*SE*	*95%CI*	*p*
**Between-level (L2)**					**Within-level (L1)**				
On-task enjoyment					On-task enjoyment				
Condition 1 vs. 3	-0.13	0.13	[-0.38, 0.12]	.314	Experimental block	0.06	0.07	[-0.08, 0.21]	.367
Condition 2 vs. 3	0.30	0.12	[0.06, 0.54]	.013	Number of hints	-0.31	0.07	[-0.44, -0.18]	.000
Condition 4 vs. 3	-0.09	0.13	[-0.33, 0.16]	.502	**Indirect effects (L2)**				
Cognitive ability	0.11	0.08	[-0.05, 0.28]	.184	Cognitive learning				
Age	-0.01	0.10	[-0.21, 0.20]	.935	Condition 1 vs. 3 → On-task enjoyment	0.01	0.02	[-0.02, 0.05]	.518
Cognitive learning					Condition 2 vs. 3 → On-task enjoyment	-0.03	0.03	[-0.08, 0.03]	.358
On-task enjoyment	-0.09	0.09	[-0.26, 0.08]	.322	Condition 4 vs. 3 → On-task enjoyment	0.01	0.02	[-0.02, 0.04]	.627
Cognitive ability	0.22	0.09	[0.04, 0.40]	.019	Task performance				
Age	-0.23	0.08	[-0.39, -0.07]	.004	Condition 1 vs. 3 → On-task enjoyment	-0.01	0.02	[-0.04, 0.02]	.532
Task performance					Condition 2 vs. 3 → On-task enjoyment	0.03	0.04	[-0.04, 0.09]	.463
On-task enjoyment	0.09	0.12	[-0.15, 0.32]	.470	Condition 3 vs. 3 → On-task enjoyment	-0.01	0.01	[-0.04, 0.02]	.603
Cognitive ability	0.25	0.07	[0.11, 0.39]	.000					
Age	-0.21	0.12	[-0.44, 0.03]	.082					

Note. Standardized results of multilevel Model $S1_{ENJOY}$, assessing indirect effects of experimental conditions on learning outcomes through on-task enjoyment. *ß* represents standardized coefficients, *SE* is the standard error, *95%CI* indicates the 95% confidence intervals, and p represents the p-value. Model fit: $\chi^2 = 16.37$, df = 12, $p = .175$, CFI = 0.94, TLI = 0.88, RMSEA = .026, $SRMR_{WITHIN} = .005$, $SRMR_{BETWEEN} = .065$.

Supplementary Table S2. Standardized results of Model $S2_{BORED}$.

	ß	*SE*	*95%CI*	*p*		*ß*	*SE*	*95%CI*	*p*
**Between-level (L2)**					**Within-level (L1)**				
On-task boredom					On-task boredom				
Condition 1 vs. 3	0.14	0.13	[-0.11, 0.39]	.281	Experimental block	0.23	0.09	[0.10, 0.35]	.001
Condition 2 vs. 3	-0.13	0.10	[-0.33, 0.08]	.217	Number of hints	0.32	0.07	[0.02, 0.21]	.023
Condition 4 vs. 3	0.24	0.12	[0.00, 0.48]	.049	**Indirect effects (L2)**				
Cognitive ability	-0.20	0.09	[-0.38, -0.01]	.038	Cognitive learning				
Age	0.04	0.09	[-0.15, 0.22]	.682	Condition 1 vs. 3 → On-task boredom	0.02	0.02	[-0.02, 0.05]	.464
Cognitive learning					Condition 2 vs. 3 → On-task boredom	-0.02	0.02	[-0.08, 0.03]	.293
On-task boredom	0.12	0.09	[-0.06, 0.30]	.182	Condition 4 vs. 3 → On-task boredom	0.03	0.03	[-0.02, 0.04]	.345
Cognitive ability	0.23	0.10	[0.04, 0.42]	.016	Task performance				
Age	-0.23	0.08	[-0.39, -0.08]	.004	Condition 1 vs. 3 → On-task boredom	-0.01	0.02	[-0.04, 0.02]	.487
Task performance					Condition 2 vs. 3 → On-task boredom	0.01	0.02	[-0.04, 0.09]	.490
On-task boredom	-0.08	0.11	[-0.29, 0.13]	.440	Condition 4 vs. 3 → On-task boredom	-0.02	0.03	[-0.04, 0.02]	.452
Cognitive ability	0.24	0.07	[0.10, 0.39]	.001					
Age	-0.21	0.12	[-0.44, 0.02]	.079					

Note. Standardized results of multilevel Model $S2_{BORED}$, assessing indirect effects of experimental conditions on learning outcomes through on-task boredom. *ß* represents standardized coefficients, *SE* is the standard error, *95%CI* indicates the 95% confidence intervals, and p represents the p-value. Model fit: $\chi^2 = 14.53$, df = 12, $p = .268$, CFI = 0.97, TLI = 0.94, RMSEA = .019, $SRMR_{WITHIN} = .005$, $SRMR_{BETWEEN} = .063$.

Supplementary Table S3. Standardized results of Model $S3_{FRUST}$.

	ß	*SE*	*95%CI*	*p*		*ß*	*SE*	*95%CI*	*p*
**Between-level (L2)**					**Within-level (L1)**				
On-task frustration					On-task frustration				
Condition 1 vs. 3	0.05	0.13	[-0.19, 0.30]	.676	Experimental block	0.03	0.09	[-0.14, 0.19]	.754
Condition 2 vs. 3	-0.08	0.12	[-0.33, 0.16]	.500	Number of hints	0.32	0.07	[0.20, 0.45]	.000
Condition 4 vs. 3	0.22	0.12	[-0.00, 0.45]	.054	**Indirect effects (L2)**				
Cognitive ability	-0.20	0.11	[-0.41, 0.01]	.062	Cognitive learning				
Age	-0.06	0.10	[-0.27, 0.14]	.559	Condition 1 vs. 3 → On-task frustration	0.01	0.02	[-0.03, 0.04]	.702
Cognitive learning					Condition 2 vs. 3 → On-task frustration	-0.01	0.02	[-0.05, 0.02]	.555
On-task frustration	0.13	0.09	[-0.05, 0.30]	.168	Condition 4 vs. 3 → On-task frustration	0.03	0.03	[-0.03, 0.08]	.318
Cognitive ability	0.23	0.09	[0.05, 0.41]	.012	Task performance				
Age	-0.22	0.08	[-0.38, -0.06]	.006	Condition 1 vs. 3 → On-task frustration	-0.01	0.02	[-0.04, 0.02]	.696
Task performance					Condition 2 vs. 3 → On-task frustration	0.01	0.01	[-0.02, 0.04]	.493
On-task frustration	-0.11	0.11	[-0.32, 0.10]	.304	Condition 4 vs. 3 → On-task frustration	-0.03	0.03	[-0.08, 0.03]	.338
Cognitive ability	0.24	0.07	[0.09, 0.38]	.001					
Age	-0.22	0.12	[-0.44, 0.01]	.061					

Note. Standardized results of multilevel Model $S3_{FRUST}$, assessing indirect effects of experimental conditions on learning outcomes through on-task frustration. *ß* represents standardized coefficients, *SE* is the standard error, *95%CI* indicates the 95% confidence intervals, and p represents the p-value. Model fit: $\chi^2 = 14.12$, df = 12, $p = .293$, CFI = 0.98, TLI = 0.95, RMSEA = .018, $SRMR_{WITHIN} = .003$, $SRMR_{BETWEEN} = .061$.

Supplementary Table S4. Standardized results of Model S4$_{\text{PLAN}}$.

	ß	*SE*	*95%CI*	*p*		*ß*	*SE*	*95%CI*	*p*
**Between-level (L2)**					**Within-level (L1)**				
Planning					Planning				
Condition 1 vs. 3	-0.17	0.08	[-0.33, -0.01]	.043	Experimental block	-0.15	0.05	[-0.25, -0.06]	.002
Condition 2 vs. 3	-0.19	0.10	[-0.39, 0.02]	.070	Number of hints	0.02	0.06	[-0.09, 0.13]	.723
Condition 4 vs. 3	-0.13	0.11	[-0.34, 0.08]	.210	**Indirect effects (L2)**				
Cognitive ability	-0.06	0.05	[-0.15, 0.03]	.212	Cognitive learning				
Age	-0.17	0.05	[-0.27, -0.07]	.001	Condition 1 vs. 3 → Planning	0.02	0.02	[-0.02, 0.05]	.396
Cognitive learning					Condition 2 vs. 3 → Planning	0.02	0.02	[-0.02, 0.06]	.407
Planning	-0.09	0.08	[-0.24, 0.06]	.240	Condition 4 vs. 3 → Planning	0.01	0.02	[-0.02, 0.05]	.505
Cognitive ability	0.20	0.09	[0.01, 0.38]	.035	Task performance				
Age	-0.24	0.08	[-0.40, -0.08]	.004	Condition 1 vs. 3 → Planning	0.02	0.02	[-0.02, 0.06]	.338
Task performance					Condition 2 vs. 3 → Planning	0.02	0.02	[-0.02, 0.06]	.288
Planning	-0.11	0.08	[-0.26, 0.04]	.159	Condition 4 vs. 3 → Planning	0.01	0.02	[-0.02, 0.05]	.443
Cognitive ability	0.26	0.08	[0.11, 0.40]	.001					
Age	-0.23	0.12	[-0.47, 0.01]	.062					

Note. Standardized results of multilevel Model S4$_{\text{PLAN}}$, assessing indirect effects of experimental conditions on learning outcomes through planning. *ß* represents standardized coefficients, *SE* is the standard error, *95%CI* indicates the 95% confidence intervals, and *p* represents the *p*-value. Model fit: $\chi^2 = 13.01$, df = 12, $p = .369$, CFI = 0.99, TLI = 0.97, RMSEA = .012, SRMR$_{\text{WITHIN}}$ = .002, SRMR$_{\text{BETWEEN}}$ = .061.

Supplementary Table S5. Standardized results of Model $S5_{MON}$.

	ß	*SE*	*95%CI*	*p*		*ß*	*SE*	*95%CI*	*p*
**Between-level (L2)**					**Within-level (L1)**				
Monitoring					Monitoring				
Condition 1 vs. 3	0.16	0.16	[-0.16, 0.48]	.280	Experimental block	-0.32	0.04	[-0.40, -0.24]	.000
Condition 2 vs. 3	0.05	0.14	[-0.25, 0.34]	.971	Number of hints	0.17	0.06	[0.06, 0.29]	.004
Condition 4 vs. 3	-0.04	0.13	[-0.31, 0.24]	.730	**Indirect effects (L2)**				
Cognitive ability	-0.12	0.11	[-0.34, 0.10]	.335	Cognitive learning				
Age	-0.25	0.12	[-0.50, -0.01]	.038	Condition 1 vs. 3 → Monitoring	-0.01	0.02	[-0.05, 0.03]	.658
Cognitive learning					Condition 2 vs. 3 → Monitoring	-0.00	0.01	[-0.02, 0.02]	.806
Monitoring	-0.05	0.12	[-0.29, 0.18]	.661	Condition 4 vs. 3 → Monitoring	0.00	0.01	[-0.02, 0.02]	.841
Cognitive ability	0.20	0.10	[0.01, 0.38]	.040	Task performance				
Age	-0.24	0.09	[-0.41, -0.06]	.007	Condition 1 vs. 3 → Monitoring	-0.03	0.03	[-0.10, 0.03]	.322
Task performance					Condition 2 vs. 3 → Monitoring	-0.01	0.03	[-0.07, 0.06]	.776
Monitoring	-0.20	0.16	[-0.51, 0.11]	.203	Condition 4 vs. 3 → Monitoring	0.01	0.03	[-0.05, 0.07]	.808
Cognitive ability	0.23	0.08	[0.08, 0.39]	.004					
Age	-0.26	0.10	[-0.46, -0.06]	.010					

Note. Standardized results of multilevel Model $S5_{MON}$, assessing indirect effects of experimental conditions on learning outcomes through monitoring. *ß* represents standardized coefficients, *SE* is the standard error, *95%CI* indicates the 95% confidence intervals, and *p* represents the *p*-value. Model fit: $\chi^2 = 14.36$, df = 12, $p = .279$, CFI = 0.98, TLI = 0.95, RMSEA = .019, $SRMR_{WITHIN} = .006$, $SRMR_{BETWEEN} = .060$.

Supplementary Table S6. Standardized results of Model $S6_{CONT/RE}$.

	ß	*SE*	*95%CI*	*p*		*ß*	*SE*	*95%CI*	*p*
**Between-level (L2)**					**Within-level (L1)**				
Control/regulation					Control/regulation				
Condition 1 vs. 3	0.09	0.11	[-0.11, 0.29]	.404	Experimental block	0.11	0.05	[0.02, 0.21]	.016
Condition 2 vs. 3	-0.11	0.12	[-0.33, 0.10]	.183	Number of hints	0.09	0.05	[-0.00, 0.18]	.061
Condition 4 vs. 3	0.10	0.17	[-0.10, 0.30]	.901	**Indirect effects (L2)**				
Cognitive ability	0.22	0.09	[0.03, 0.41]	.014	Cognitive learning				
Age	-0.38	0.10	[-0.59, -0.18]	.000	Condition 1 vs. 3 → Control/regulation	0.06	0.07	[-0.07, 0.19]	.400
Cognitive learning					Condition 2 vs. 3 → Control/regulation	-0.07	0.07	[-0.20, 0.06]	.305
Control/regulation	0.63	0.10	[0.43, 0.83]	.000	Condition 4 vs. 3 → Control/regulation	0.06	0.07	[-0.07, 0.20]	.351
Cognitive ability	0.08	0.09	[-0.09, 0.24]	.373	Task performance				
Age	0.01	0.09	[-0.17, 0.18]	.953	Condition 1 vs. 3 → Control/regulation	0.08	0.09	[-0.10, 0.26]	.391
Task performance					Condition 2 vs. 3 → Control/regulation	-0.10	0.09	[-0.28, 0.09]	.291
Control/regulation	0.89	0.08	[0.74, 1.04]	.000	Condition 4 vs. 3 → Control/regulation	0.09	0.10	[-0.10, 0.27]	.348
Cognitive ability	0.08	0.07	[-0.06, 0.22]	.264					
Age	0.11	0.09	[-0.07, 0.29]	.214					

Note. Standardized results of multilevel Model $S6_{CONT/RE}$, assessing indirect effects of experimental conditions on learning outcomes through control/regulation. *ß* represents standardized coefficients, *SE* is the standard error, *95%CI* indicates the 95% confidence intervals, and p represents the p-value. Model fit: $\chi^2 = 13.86$, df = 12, $p = .310$, CFI = 0.99, TLI = 0.97, RMSEA = .017, $SRMR_{WITHIN} = .005$, $SRMR_{BETWEEN} = .062$.

Supplementary Table S7. Standardized results of Model S7$_{REFL/EV}$.

	ß	*SE*	*95%CI*	*p*		*ß*	*SE*	*95%CI*	*p*
**Between-level (L2)**					**Within-level (L1)**				
Reflection/evaluation					Reflection/evaluation				
Condition 1 vs. 3	0.12	0.15	[-0.17, 0.40]	.427	Experimental block	0.08	0.05	[-0.01, 0.17]	.095
Condition 2 vs. 3	-0.01	0.14	[-0.28, 0.28]	.974	Number of hints	0.08	0.05	[-0.02, 0.17]	.128
Condition 4 vs. 3	0.38	0.13	[0.12, 0.64]	.004	**Indirect effects (L2)**				
Cognitive ability	-0.15	0.18	[-0.49, 0.20]	.400	Cognitive learning				
Age	0.09	0.12	[-0.15, 0.33]	.472	Condition 1 vs. 3 → Reflection/evaluation	0.06	0.08	[-0.09, 0.20]	.466
Cognitive learning					Condition 2 vs. 3 → Reflection/evaluation	-0.00	0.07	[-0.14, 0.13]	.974
Reflection/evaluation	0.48	0.14	[0.20, 0.75]	.001	Condition 4 vs. 3 → Reflection/evaluation	0.18	0.07	[0.04, 0.32]	.012
Cognitive ability	0.27	0.11	[0.07, 0.48]	.010	Task performance				
Age	-0.28	0.08	[-0.44, -0.11]	.001	Condition 1 vs. 3 → Reflection/evaluation	0.04	0.06	[-0.07, 0.15]	.483
Task performance					Condition 2 vs. 3 → Reflection/evaluation	-0.00	0.05	[-0.10, 0.09]	.974
Reflection/evaluation	0.34	0.11	[0.12, 0.56]	.003	Condition 4 vs. 3 → Reflection/evaluation	0.13	0.06	[0.02, 0.24]	.022
Cognitive ability	0.31	0.08	[0.16, 0.46]	.000					
Age	-0.25	0.11	[-0.46, -0.03]	.026					

Note. Standardized results of multilevel Model S7$_{REFL/EV}$, assessing indirect effects of experimental conditions on learning outcomes through reflection/evaluation. *ß* represents standardized coefficients, *SE* is the standard error, *95%CI* indicates the 95% confidence intervals, and *p* represents the *p*-value. Model fit: $\chi^2 = 9.08$, df = 12, $p = .697$, CFI = 1.00, TLI = 1.00, RMSEA <. 001, SRMR$_{WITHIN}$ = .013, SRMR$_{BETWEEN}$ = .051.
